# Clinical features and serum cytokine profiles of elderly-onset adult-onset Still’s disease

**DOI:** 10.1038/s41598-022-25514-6

**Published:** 2022-12-09

**Authors:** Mizuki Yagishita, Hiroto Tsuboi, Yuki Kuroda, Tomonori Sawabe, Akira Kawashima, Fumina Kawashima, Nana Uematsu, Ryota Sato, Taihei Nishiyama, Mayu Terasaki, Hirofumi Toko, Fumika Honda, Ayako Ohyama, Saori Abe, Ayako Kitada, Haruka Miki, Shinya Hagiwara, Yuya Kondo, Takayuki Sumida, Isao Matsumoto

**Affiliations:** Department of Rheumatology, Faculty of Medicine, University of Tsukuba, 1-1-1 Tennodai, Tsukuba, Ibaraki 305-8575 Japan

**Keywords:** Rheumatology, Rheumatic diseases

## Abstract

Recent studies have suggested that the clinical features of elderly-onset adult-onset Still’s disease (AOSD) differ from those of young and middle-aged-onset patients, whereas the details remain unclear, and cytokine profiles of elderly-onset AOSD have not been reported. To clarify the clinical features and cytokine profiles of elderly-onset AOSD, we examined patients with AOSD who developed the disease between January 2006 and September 2021. We divided the patients into the young and middle-aged-onset group (aged < 65 years) and the elderly-onset group (aged ≥ 65 years) and compared the groups in terms of patient characteristics, clinical symptoms, laboratory findings including serum interleukin (IL)-6 and IL-18, treatment, and prognosis. A total of 48 patients were examined (10 in the elderly-onset group). In the elderly-onset group, atypical rash was significantly more frequent, typical rash and splenomegaly were significantly less frequent, white blood cell count and neutrophil ratio were significantly higher and serum IL-6 levels were significantly lower. Serum IL-6 showed a significantly negative correlation with age at onset. Treatment and relapse were comparable between the 2 groups, whereas infections were significantly more frequent in the elderly-onset group. The clinical features and cytokine profiles of elderly-onset AOSD might differ from those of young and middle-aged-onset AOSD.

## Introduction

Adult-onset Still’s disease (AOSD) is a systemic inflammatory disorder characterized by symptoms including rash, spiking fever, arthritis, sore throat, and hepatosplenomegaly. Regarding the age of onset, it has been conventionally considered that AOSD typically occurs in young people in their 20 s or 30 s^1^. Indeed, reports in the 1990s indicated that the mean age of the disease onset was in the 30 s^2,3^. In recent years, however, an increase in age of disease onset has been noted, with the mean in the 40 s and 50 s^4,5^. In addition, it is not uncommon to find cases of AOSD in the elderly population, those aged 65 years or older. Recently, attention has been focused on the possibility that the clinical characteristics of these patients with elderly-onset AOSD might differ from those of patients who develop the disease in early adulthood or in middle age. Several clinical features of elderly-onset AOSD have been reported, including a less typical skin rash and more disseminated intravascular coagulation (DIC)^6–8^. However, few studies have been conducted to date, and the clinical features of the disease have not yet been fully clarified.

One of the characteristic features of the laboratory findings for AOSD is a marked increase in serum ferritin. Inflammatory cytokines are known to play important roles in the pathogenesis of AOSD, and the increase in serum ferritin is thought to reflect a cytokine storm^9^. Interleukin (IL)-1β, IL-6, and IL-18 are cytokines known to be involved in the pathogenesis of AOSD, and each has been reported to be associated with the clinical findings of AOSD. For example, IL-1β has been reported to be associated with systemic symptoms such as fever; IL-6, with arthritis and typical skin rash; and IL-18, with hemophagocytic syndrome and liver injury^10–14^. These reports suggested that the clinical phenotype of AOSD might be associated with the cytokine profiles. Therefore, it is conceivable that the aforementioned differences in clinical features between elderly-onset AOSD and young and middle-aged-onset AOSD could be related to differences in the cytokine profiles. However, no reports have been published on an association between age of onset and the cytokine profile in AOSD.

In this study, we focused on the serum cytokines of AOSD and aimed to clarify the clinical features and cytokine profiles of elderly-onset AOSD patients in comparison with those of young and middle-aged-onset AOSD patients.

## Results

### Characteristics and clinical presentations

**Figure 1:**
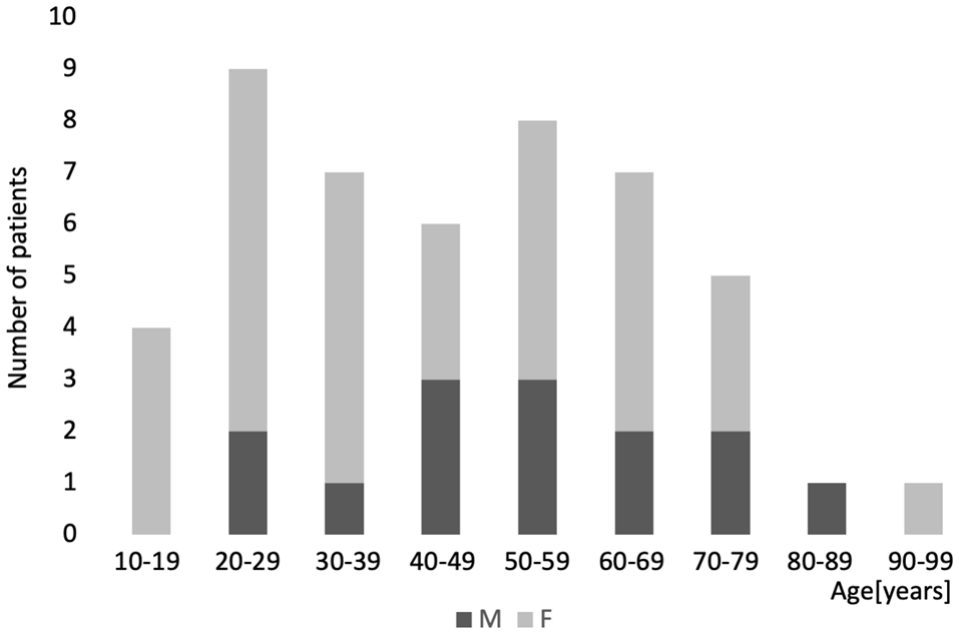
Distribution of age at onset and of sex. The distribution was bimodal, with peaks in the 20 s and 50 s.

A total of 48 patients with AOSD definitively diagnosed on the basis of the Yamaguchi criteria were enrolled in this study; their median age was 47.5 (interquartile range 28.3–61.8) years, and 14 of them were male. The patients’ characteristics and clinical presentations are detailed in Table 1. The distribution by age at onset and sex is shown in the Fig. 1. A bimodal distribution with peaks in the 20 s and 50 s was found. The young and middle-aged-onset group comprised 38 cases, and the elderly-onset group, 10 cases; the ages at onset of the groups were 38.0 (24.8–51.5) years and 73.0 (68.8–79.8) years, respectively. No significant differences between the 2 groups were found in sex, time from onset to initiation of treatment, observation period, or complications from other autoimmune diseases.

**Table 1:** Comparison of the characteristics and clinical presentations of the young and middle-aged-onset and elderly-onset groups.

	Young and middle-aged-onset	Elderly-onset	*p*-value
	n = 38	n = 10	
**Profiles**			
Age at onset, y	38.0 (24.8–51.5)	73.0 (68.8–79.8)	< 0.01**
Sex	M10:F28	M4:F6	0.45
Time from onset to treatment, wk	4.8 (3.5–11.4)	5.6 (2.8–7.3)	0.57
Observation period, mo	32.5 (11.5–66.5)	14.5 (2.0–34.0)	0.18
**Clinical features**			
Fever	38 (100.0%)	10 (100.0%)	1.00
Arthritis	30 (77.8%)	8 (80.0%)	1.00
Typical rash	26 (68.4%)	2 (20.0%)	0.02*
Atypical rash	9 (23.7%)	10 (100.0%)	< 0.01**
Sore throat	26 (68.4%)	8 (80.0%)	0.70
Lymphadenopathy	22 (57.9%)	3 (30.0%)	0.16
Splenomegaly	26 (68.4%)	0 (0.0%)	< 0.01**
Serositis	7 (18.4%)	1 (10.0%)	1.00
Number of items fitting Yamaguchi criteria^a^	7.0 (6.0–7.0)	6.0 (5.0–6.0)	< 0.01**
Severity score^b^	3.0 (2.0–4.0)	3.0 (2.0–4.3)	0.65
**Complications**			
HLH	2 (5.3%)	2 (20.0%)	0.59
DIC	5 (13.2%)	1(10.0%)	1.00
Alveolar hemorrhage	1 (2.6%)	0 (0.0%)	1.00
Concomitant autoimmune diseases	2 (5.3%)	0 (0.0%)	1.00

*HLH* hemophagocytic lymphohistiocytosis, *DIC* disseminated intravascular coagulation.

Data represent the median (interquartile range) or numbers of patients.

^a^8 items in total: 4 major items and 4 minor items.

^b^0–9 points in total (1 point: serositis, neutrophil ratio ≥ 85%, ferritin ≥ 3000 ng/mL, marked lymphadenopathy and resistance to steroid therapy; 2 points: DIC and HLH).

*p*-value: Fisher exact test for categorical variables and Mann–Whitney U test for continuous variables.

*Significant value (*p* < 0.05).

**Significant value (*p* < 0.01).

In the young and middle-aged-onset group, typical skin rash was more frequent (68.4%), whilst atypical skin rash was less frequent (23.7%). In contrast, in the elderly-onset group, only 20.0% of the patients had a typical skin rash, and an atypical skin rash was observed in all the patients. The frequency of both types of skin rash differed significantly between the 2 groups. No significant difference in lymphadenopathy was found between the groups, but splenomegaly was significantly less frequent in the elderly-onset group (68.4% vs. 0.0%, *p* < 0.01). No significant differences in Hemophagocytic lymphohistiocytosis (HLH) or DIC complications were found between the 2 groups. The number of items applicable to the Yamaguchi criteria (8 items in total: 4 major items and 4 minor items) was significantly lower in the elderly-onset group (7.0 [6.0–7.0] vs. 6.0 [5.0–6.0], *p* < 0.01) (Table 1).

### Laboratory data and cytokines

**Figure 2:**
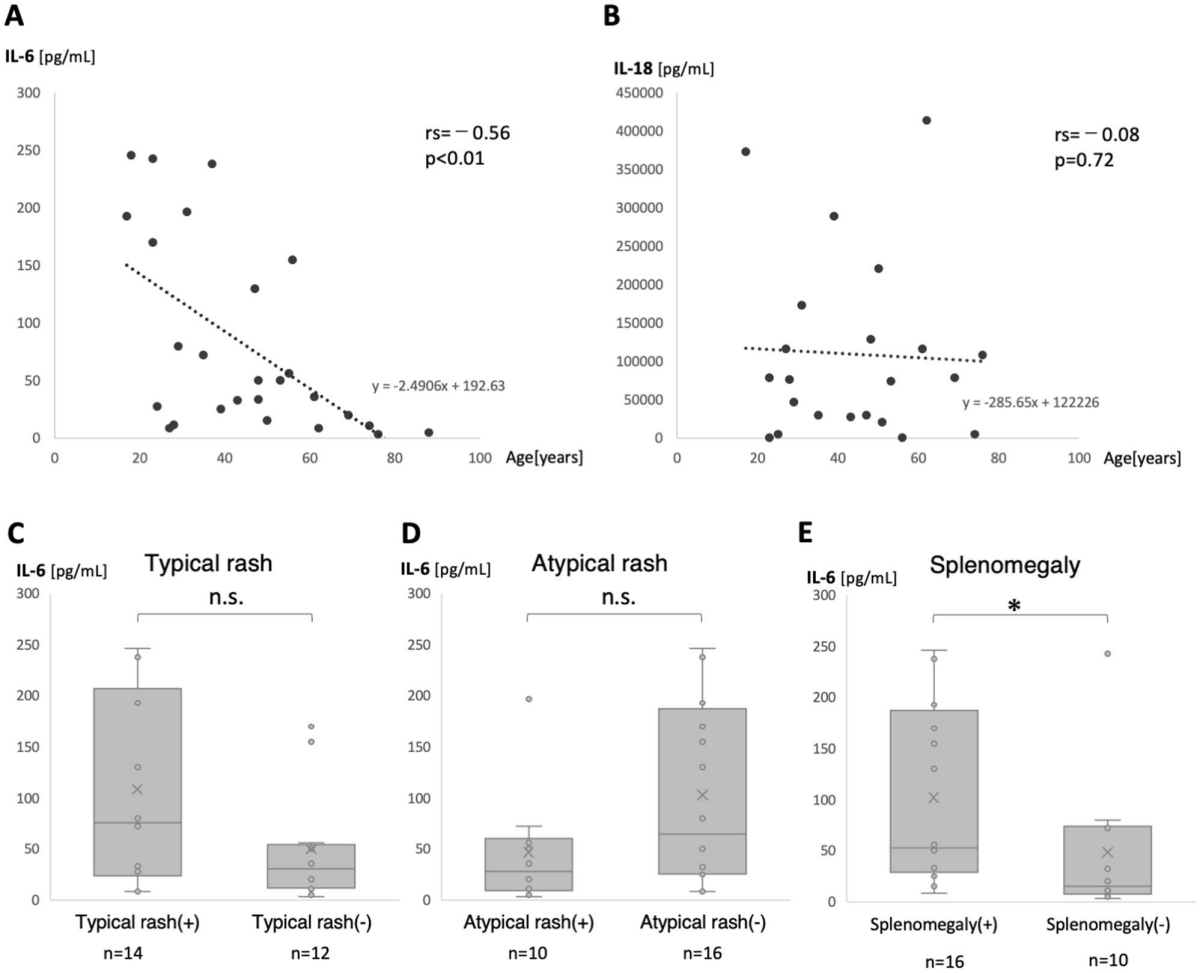
Correlation between cytokines and age at onset and between IL-6 and clinical manifestations. (**A**) Serum IL-6 showed a significant negative correlation with age (n = 26). (**B**) Serum IL-18 showed no significant correlation with age (n = 22). (**C**) Serum IL-6 levels tended to be higher in patients with typical skin rash than in those without, although the difference was not significant (*p* = 0.12). (**D**) Serum IL-6 levels tended to be lower in patients with atypical skin rash than in those without, although the difference was not significant (*p* = 0.14). (**E**) Serum IL-6 levels were significantly higher in patients with splenomegaly than in those without (*p* = 0.04). (**A**), (**B)**: *p*-values were assessed by use of the Spearman rank correlation coefficient between age and serum cytokine concentration. (**C**)–(**E**): *p*-values were assessed by use of the Mann–Whitney U test to compare the 2 groups. n.s. not significant; *p < 0.05.

The white blood cell (WBC) count (12,400 [10,600–18,150] vs 19,450 [16,325–23,600]/μL, *p* = 0.04) and neutrophil ratio (88.0 [83.4–91.0] vs. 94.9 [90.8–97.2] %, *p* < 0.01) were significantly higher in the elderly-onset group. No significant differences in CRP or ferritin levels were found between the 2 groups. Serum IL-6 before the initiation of treatment in the elderly-onset group was 7.75 (3.6–17.7, n = 4), which was significantly lower than that in the young and middle-aged-onset group (53.2 [27.0–175.8], n = 22, *p* < 0.01). No significant difference in serum IL-18 was found between the 2 groups (Table 2). Interestingly, serum IL-6 showed a significantly negative correlation with age at onset, whilst no correlation with age was observed for serum IL-18 (Fig. 2A,B). The correlation between HLH complications and serum cytokines was also examined, regardless of age of onset. A total of 4 patients had concomitant HLH, of whom IL-6 was measured in 3 patients and IL-18 in only 1 patient. There were no significant differences in these cytokines between patients with and without HLH (Supplementary Fig. S1).

**Table 2:** Comparison of the laboratory data and cytokine profiles of the young and middle-aged-onset and elderly-onset groups.

	Young and middle-aged-onset	Elderly-onset	*p*-value
	n = 38	n = 10	
WBC (μL)	12,400 (10,600–18,150)	19,450 (16,325–23,600)	0.04*
Neutrophil (%)	88.0 (83.4–91.0)	94.9 (90.8–97.2)	< 0.01**
Hb (mg/dL)	11.1 (9.9–11.9)	11.3 (9.9–12.3)	0.66
Plt, × 10^4^/μL	31.7 (24.4–39.4)	27.2 (21.1–33.0)	0.24
AST, IU/L	49.0 (37.8–115.0)	71.0 (59.8–96.0)	0.79
ALT, IU/L	50.0 (23.5–93.8)	47.5 (32.0–154.5)	0.19
LDH, IU/L	502.5 (363.0–994.3)	466.0 (396.3–628.8)	0.33
CRP (mg/dL)	10.8 (7.1–17.3)	17.3 (9.0–20.3)	0.53
Ferritin (ng/mL)	7632 (2,745–18,495)	8,375 (3796–22,128)	0.82
sIL2R (U/mL)	1633 (1,120–2,077)	1180 (896–1,715)	0.18
ANA-positive	7 (18.4%)	3 (30.0%)	0.41
RF-positive	2 (5.2%)	1 (10.0%)	0.51
RF titer, IU/mL	3.0 (2.0–5.0)	2.5 (2.0–8.8)	0.68
IL-6 (pg/mL)	53.2 (27.0–175.8)	7.75 (3.6–17.7)	< 0.01**
	(n = 22)	(n = 4)	
IL-18 (pg/m)	76,200 (27,200–173,000)	78,900 (5000–108,000)	0.70
	(n = 19)	(n = 3)	

*ANA* antinuclear antibody.

Data represent the median (interquartile range) or numbers of patients.

*p*-value: Fisher exact test for categorical variables and Mann–Whitney U test for continuous variables.

*Significant value (*p* < 0.05).

**Significant value (*p* < 0.01).

### Correlation between serum IL-6 levels and clinical manifestations

The correlations of serum IL-6 levels, which differed significantly between the young and middle-aged-onset group and the elderly-onset group, with the clinical manifestations, including typical or atypical rash, WBC count and neutrophil ratio, and splenomegaly, were analyzed. Although no significant differences were found in the serum IL-6 levels between patients with and without typical rashes or atypical rashes, the levels tended to be higher in the patients with typical skin rashes (76.2 [23.5–207.3] vs. 30.4 [12.2–54.6] pg/mL, *p* = 0.12) and lower in those with atypical skin rashes (27.9 [9.2–60.2] vs. 64.9 [25.7–197.3] pg/mL, *p* = 0.14). As for splenomegaly, serum IL-6 levels were significantly higher in the patients with the lesions than in those without the lesions (53.2 [29.1–187.3] vs. 15.6 [7.4–74.4] pg/mL, *p* = 0.04) (Fig. 2C–E). The Spearman rank correlation coefficient showed no significant correlation between serum IL-6 levels and WBC count (rs = −0.31, *p* = 0.13) or neutrophil ratio (rs = −0.24, *p* = 0.23).

### Pathology of skin rash and bone marrow

Skin biopsy was performed in 70.0% of the elderly-onset group and in 34.2% of the young and middle-aged-onset group, with no significant difference in the rate of skin biopsy. Superficial perivascular dermatitis was observed in 84.6% of the young and middle-aged-onset group and in 14.3% of the elderly-onset group, and was significantly less frequent in the latter group (*p* < 0.01). On the other hand, interface dermatitis was significantly more frequent in the elderly-onset group (15.4% vs. 71.4%, *p* = 0.02; Table 3).

**Table 3:** Comparison of the pathological findings of the skin biopsy and bone marrow of the young and middle-aged-onset and elderly-onset groups.

		Young and middle-aged-onset	Elderly-onset	*p*-value
		n = 38	n = 10	
**Skin biopsy**		13 (34.2%)	7 (70.0%)	0.07
Superficial perivascular dermatitis		11/13 (84.6%)	1/7 (14.3%)	< 0.01**
Interface dermatitis		2/13 (15.4%)	5/7 (71.4%)	0.02*
Spongiotic dermatitis		0/13 (0.0%)	1/7 (14.3%)	0.38
**Bone marrow examination**		11 (28.9%)	3 (30.0%)	1.00
Hemophagocytic macrophages		1/11 (9.0%)	1/3 (33.3%)	0.40

Data represent the number of patients.

*p*-value: Fisher exact test for categorical variables.

*Significant value (*p* < 0.05).

**Significant value (*p* < 0.01).

Bone marrow examination was performed in 30.0% of the elderly-onset group and in 28.9% of the young and middle-aged-onset group. Phagocytosis was found in 9.0% of the young and middle-aged-onset group and in 33.3% of the elderly-onset group, with no significant difference (Table 3).

### Treatment and prognosis

No significant differences between the 2 groups were found in terms of initial prednisolone (PSL) dose, steroid pulse therapy, concomitant immunosuppressive agents and tocilizumab, or therapeutic plasma exchange. No differences between the 2 groups were found in resistance to steroid therapy (impossibility to taper prednisolone to < 0.4 mg/kg/day, because of residual disease activity of AOSD), discontinuation of treatment, or relapse after treatment. Tocilizumab was administered to 6 cases in the young and middle-aged-onset group and 1 case in the elderly-onset group. There was no difference in efficacy between the two groups, as all patients treated with tocilizumab were able to reduce PSL without relapse. The prevalence of concomitant infection within a year of treatment was significantly higher in the elderly-onset group (23.6% vs. 80.0%, *p* < 0.01). In the young and middle-aged-onset group, 5 cases of CMV antigenemia and 1 case each of cellulitis, *Candida* infection, herpes zoster, and sepsis were observed. In the elderly-onset group, CMV antigenemia was frequently observed, in 8 cases (80.0%), and pneumocystis pneumonia (PCP) was observed in 2 cases, and bronchopneumonia, in 1 case. No significant difference in death during the observation period was found between the 2 groups. Fatal cases included 1 case of sepsis in the young and middle-aged-onset group and 1 case of PCP in the elderly-onset group (Table 4).

**Table 4:** Comparison of the treatment and prognosis of the young and middle-aged-onset and elderly-onset groups.

	Young and middle-aged-onset	Elderly-onset	*p*-value
	n = 38	n = 10	
**Treatment**			
Initial dose of prednisolone, mg/day	50.0 (40.0–60.0)	37.5 (25.0–60.0)	0.13
Methylprednisolone pulse therapy	16 (42.1%)	4 (40.0%)	1.00
Immunosuppressants	14 (36.8%)	2 (20.0%)	0.45
Tocilizumab	6 (15.8%)	1 (10.0%)	1.00
Therapeutic plasma exchange	2 (5.3%)	1 (10.0%)	0.55
**Prognosis**			
Steroid-resistant*	19 (50.0%)	4 (40.0%)	0.73
Discontinuation of treatment	6 (15.8%)	0 (0.0%)	0.30
From initiation to discontinuation of treatment, mo	13.5 (5.5–53.0)	–	–
Relapse after treatment	10 (26.3%)	1 (10.0%)	0.42
From initiation of treatment to relapse, mo	12.5 (7.3–19.5)	7	0.34
Concomitant infection within a year	9 (23.6%)	8 (80.0%)	< 0.01**
Death	1 (2.6%)	1 (10.0%)	0.37

Data represent median (interquartile range) or numbers of patients.

*Steroid-resistant; impossibility to taper prednisolone to < 0.4 mg/kg/day because of residual disease activity of AOSD.

*p*-value: Fisher exact test for categorical variables and Mann–Whitney U test for continuous variables.

**Significant value (*p* < 0.01).

## Discussion

In this study, 48 AOSD patients were divided into an elderly-onset group (10 patients) and a young and middle-aged-onset group (38 patients) for retrospective comparison. The elderly-onset group had significantly fewer cases of typical skin rash and splenomegaly and a low number of items applicable to the Yamaguchi criteria, and significantly more cases of atypical skin rash. The WBC count and neutrophil ratio were significantly higher in the elderly-onset group, and serum IL-6 was significantly lower in the group, showing a significant negative correlation with age. In the elderly-onset group, the pathology of skin rash showed significantly less superficial perivascular dermatitis and significantly more frequent interface dermatitis. The frequency of infectious complications was significantly higher in the elderly-onset group, with 80% of that group developing CMV antigenemia. We focused on 3 important findings as follows.

First, we noted differences in the characteristics of the clinical presentations. The young and middle-aged-onset group and elderly-onset group differed significantly in terms of the clinical symptoms including typical skin rash, atypical skin rash, and splenomegaly. In addition, the WBC count and neutrophil ratio were significantly higher in the elderly-onset group. Previous studies have reported that the clinical characteristic of elderly-onset AOSD included less typical skin rash^6^, which was consistent with the results of the present study. Another study reported significantly less splenomegaly in the elderly-onset group^15^, which was also similar to the findings of the present study. Several characteristics in the elderly-onset AOSD including more frequent DIC and higher ferritin have been commonly reported in previous studies^7,8,15^. The characteristics of more frequent serositis and higher risk of death reported in recent studies with more than 200 cases were also consistent with previous reports^16,17^. However, these characteristics were not observed in our study, which may be owing to the relatively small number of our cases. The pathological findings from the skin biopsies also differed between the 2 groups of our study. This difference might be related to the difference in the frequency of typical and atypical skin rashes between the 2 groups. Although the pathological findings of skin rashes in AOSD are generally nonspecific, superficial perivascular dermatitis was previously often observed in typical skin rashes^18–20^. On the other hand, interface dermatitis tends to be observed in atypical skin rashes, making it more important to distinguish between drug eruptions and other types of dermatitis^20–22^. In this study, interface dermatitis was significantly more frequent and superficial perivascular dermatitis was significantly less frequent in the elderly-onset group, which was consistent with the clinical features showing that atypical skin rash was more common and typical skin rash was less common in the elderly-onset group. Furthermore, the number of items applicable to the Yamaguchi criteria was significantly lower in the elderly-onset group, indicating that elderly-onset AOSD might present an atypical clinical phenotype. Therefore, when AOSD is suspected in elderly patients, more careful examination, including skin biopsy and bone marrow examination, should be considered to confirm the diagnosis of AOSD.

The second important finding is the difference in the characteristics of the serum cytokines. Serum IL-6 levels were significantly lower in the elderly-onset group and negatively correlated with age at onset, which is, to our knowledge, the first-reported such finding. In healthy individuals, serum IL-6 concentrations were reportedly higher in elderly individuals than in the young^23^. The present study’s findings on the relationship between IL-6 and age were opposite to those for healthy individuals, indicating that the finding might be derived from the pathophysiological characteristics of AOSD. Although it is not clear the mechanism of why IL-6 is lower in elderly patients with AOSD, the following reports may be helpful in considering this issue. According to a report on cytokine production of monocytes and peripheral blood mononuclear cells (PBMCs) in healthy subjects, the patterns of cytokine production in response to lipopolysaccharide (LPS) stimulation was different between young and elderly subjects in PBMCs, but not different in monocytes^24^. This suggests that lymphocytes may influence the differences between young and elderly subjects in cytokine responses of macrophage to antigen stimulation. In another report, IL-6 production from PBMCs were examined in healthy young and elderly subjects^25^. PBMCs were cultured in autologous plasma or fetal bovine serum and stimulated with phytohemagglutinin (PHA) or concanavalin A (ConA); differences in IL-6 production between young and elderly subjects were observed only under the condition of a combination of autologous plasma and stimulation with ConA. In that condition, the elderly subjects presented lower IL-6 production than the young, similar to the findings we observed in AOSD patients in our study. Based on these reports, one possible explanation for the lower IL-6 levels in the elderly-onset group is that IL-6 production in the elderly may be lower than in the young under certain environment including lymphocyte involvement or certain kind of antigen, and we speculate that the immunological conditions of AOSD may be similar to such environment. A relationship between cytokines and age of disease onset has been reported for rheumatoid arthritis (RA), whereas it has never been reported for AOSD^26^. The clinical features of elderly-onset RA are known to differ from those of young- and middle aged-onset cases, such as acute onset and frequent involvement of large arthritis. According to that study, elderly-onset RA patients had significantly higher levels of serum IL-6 and lower levels of TNF-α than those of young- and middle aged-onset patients, suggesting that age-related cytokine characteristics might be responsible for the differences in the clinical features. As for AOSD, our study found a negative correlation between age of disease onset and serum IL-6, suggesting that differences in cytokine characteristics could be related to the differences in clinical features between the 2 groups described above. In the present study, serum IL-6 tended to be higher in patients with typical skin rash, and similar results have been previously reported^12^. Furthermore, our results showed that serum IL-6 tended to be lower in patients with atypical skin rash and was significantly higher in patients with splenomegaly than in those without. These results support the hypothesis that the cytokine profiles that vary with age of AOSD onset influence the differences in the clinical characteristics, including types of skin rash and splenomegaly. In addition, HLH or macrophage activation syndrome (MAS) is an important complication of AOSD in terms of cytokine involvement, as it is known to cause cytokine storms. It has been reported that cytokine profiles differ in AOSD depending on whether MAS is complicated or not^27^. However, the number of patients with HLH/MAS was small in our study, and we could not demonstrate significant relationship between HLH/MAS and cytokines.

The third important finding is the complication of infection after treatment. Although not limited to AOSD, infectious diseases are often a problem when aggressive immunosuppressive therapy is given to elderly patients. AOSD patients are frequently treated with immunosuppressive agents or biologics in addition to high-dose corticosteroid, which may increase the risk of infectious complications. In a previous report on the clinical characteristics of elderly-onset AOSD, a higher frequency of infectious complications was reported in the elderly-onset group than in the young and middle-aged-onset group^6^. Similarly, in our study, infectious complications after the treatment were significantly higher in the elderly-onset group. Especially, CMV antigenemia was found in 80% of the elderly-onset group. A similar finding of a high prevalence of CMV infection in elderly-onset AOSD has been previously reported^8^. We considered that careful observation, including monitoring for opportunistic infections, is important when initiating treatment, especially for elderly-onset AOSD.

Our study has several limitations. First, multivariate analysis could not be performed owing to the insufficient number of patients. Second, serum cytokines were not measured in all the patients, and the number of cases for analysis was small. In addition, only IL-6 and IL-18 cytokines were measured, not IL-1β and other cytokines thought to be involved in the pathogenesis of AOSD. Further studies are needed with larger numbers of cases and more types of cytokines. Finally, the mechanism of the difference in cytokine characteristics between the two groups is not clear. Therefore, transcriptome analysis by RNA-sequencing on monocytes, which are considered to play a central role in cytokine production in AOSD, might be helpful to clarify the differences in gene expression between the two groups and identify molecules associated with age of disease onset. Comparison of immune cell subsets including lymphocytes might also be needed to clarify whether the immune cell environment differs between the two groups.

In conclusion, our study suggested that the clinical features and serum cytokine profiles of elderly-onset AOSD might differ from those of young and middle-aged-onset AOSD, which might affect the diagnostic and therapeutic strategies for elderly-onset AOSD.

## Patients and methods

### Patients

We retrospectively reviewed the charts of patients with AOSD diagnosed sometime between January 2006 and September 2021 at the University of Tsukuba Hospital (Ibaraki, Japan). A definitive diagnosis of AOSD was made on the basis of the Yamaguchi criteria^28^. We defined the elderly-onset group as patients who developed AOSD when aged 65 years or older and the young and middle-aged-onset group as patients who developed the disease when aged younger than 65 years.

Approval for this study was obtained from the Clinical Research Ethics Review Committee, University of Tsukuba Hospital (approval number: H29-154). All methods were performed in accordance with the relevant guidelines and regulations. With the approval of the Clinical Research Ethics Review Committee, University of Tsukuba Hospital, the need for written informed consent was waived by the opt-out method on the website of University of Tsukuba Hospital (https://tsukuba-rheumatology.jp/) because of the retrospective and observational design that used only clinical data obtained through daily clinical practice.

### Data collection

We retrospectively investigated the patient characteristics, clinical symptoms, laboratory findings including serum IL-6 and IL-18, treatment, and prognosis. Moreover, we performed comparison of these parameters between the elderly-onset group and the young and middle-aged-onset group.

Peripheral blood samples were taken before the initiation of treatment. Serum IL-6 concentrations were measured by chemiluminescent enzyme immunoassays (CLEIA) (normal range ≤ 2.41 pg/mL) and serum IL-18 by fluorescence enzyme immunoassay (FEIA) (normal range: not available). A typical skin rash was defined as a rash that appeared and disappeared along with fever; an atypical skin rash was defined as persistent erythema clinically considered to be caused by AOSD rather than by other factors such as infections or drugs. Splenomegaly and lymphadenopathy were diagnosed by use of computed tomography (CT). HLH was diagnosed according to the HLH-2004 guidelines^29^. The definition of DIC was based on the diagnostic criteria of the Japanese Society on Thrombosis and Hemostasis^30^. The severity of AOSD was assessed using a severity score proposed by the Ministry of Health, Labour and Welfare of Japan. The score is from 0 to 9 points in total, with 1 point each for serositis, neutrophil ratio ≥ 85%, ferritin ≥ 3000 ng/mL, marked lymphadenopathy, and resistance to steroid therapy (prednisolone ≥ 0.4 mg/kg), and 2 points each for DIC and HLH. Patients with a total score of 3 or more are regarded as severe cases; those with a total score of 2, as moderate severity cases; and those with a total score of 1 or less, as mild cases (https://www.nanbyou.or.jp/entry/282).

Cytomegalovirus (CMV) antigenemia without signs of infection was included as infection after treatment.

### Statistical analyses

For comparison of the 2 groups, the Fisher exact test was used for categorical variables, and the Mann–Whitney U test, for continuous variables. For analysis of the relationship between age and serum cytokine concentration, the Spearman tank correlation coefficient was used. Probability values of less than 0.05 were considered significant.

## Supplementary Information

Supplementary Information.

## Data Availability

The datasets used and analyzed during the current study are available from the corresponding author upon reasonable request.
