# Supplementary material for: Clinical features and serum cytokine profiles of elderly-onset adult-onset Still’s disease
Source: Sci Rep. 2022 Dec 9;12:21334. doi: 10.1038/s41598-022-25514-6 (PMC9734186; doi:10.1038/s41598-022-25514-6)
Supplement: Supplementary file 1 — Supplementary Information. [file 41598_2022_25514_MOESM1_ESM.docx]

**Supplementary Information**

**Clinical features and serum cytokine profiles of elderly-onset adult-onset Still’s disease**

Mizuki Yagishita, Hiroto Tsuboi, Yuki Kuroda, Tomoki Sawabe, Akira Kawashima, Fumina Kawashima, Nana Uematsu, Ryota Sato, Taihei Nishiyama, Mayu Terasaki, Hirofumi Toko, Fumika Honda, Ayako Ohyama, Saori Abe, Ayako Kitada, Haruka Miki, Shinya Hagiwara, Yuya Kondo, Takayuki Sumida, and Isao Matsumoto

Department of Rheumatology, Faculty of Medicine, University of Tsukuba


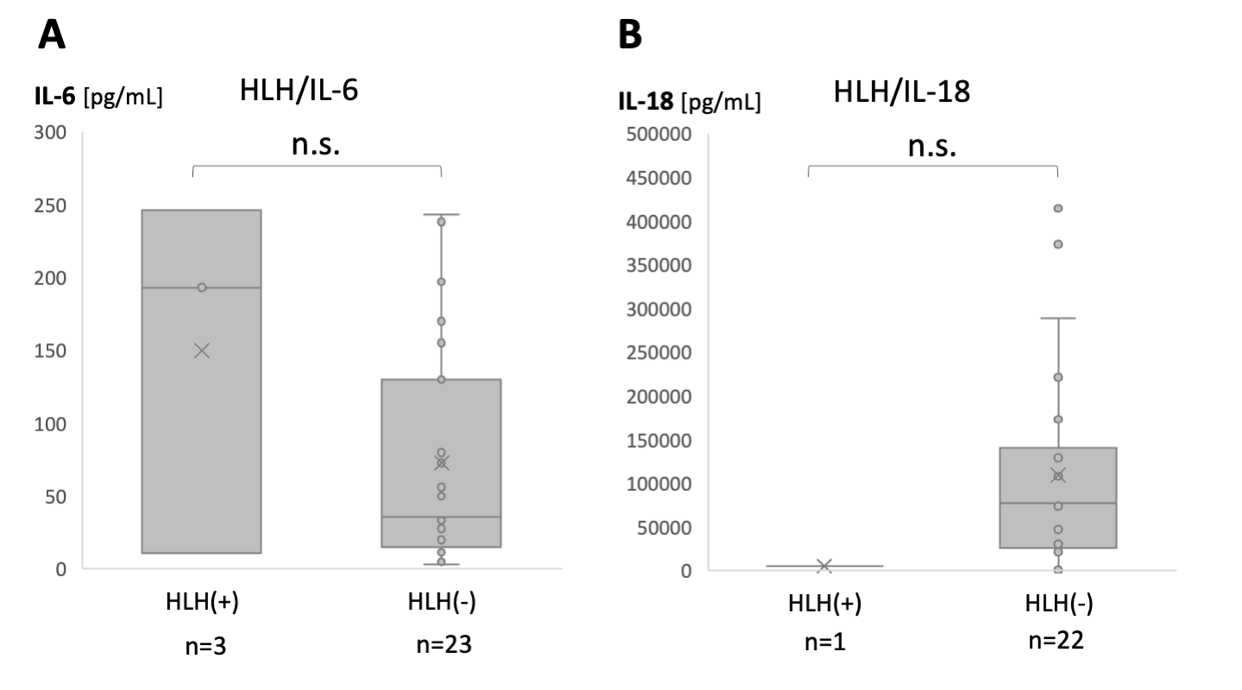


**Supplementary Figure S1. Correlation between cytokines and HLH**

1. IL-6 levels were not significantly different between patients with and without HLH (p=0.32).
2. IL-18 levels were not significantly different between patients with and without HLH (p=0.23).
